# Supplementary material for: Real-World Comparison of Trifluridine–Tipiracil with or Without Bevacizumab in Patients with Refractory Metastatic Colorectal Cancer
Source: Biomedicines. 2025 Apr 16;13(4):976. doi: 10.3390/biomedicines13040976 (PMC12024628; doi:10.3390/biomedicines13040976)
Supplement: Supplementary file 1 [file biomedicines-13-00976-s001.zip › biomedicines-3572182-supplementary.pdf]

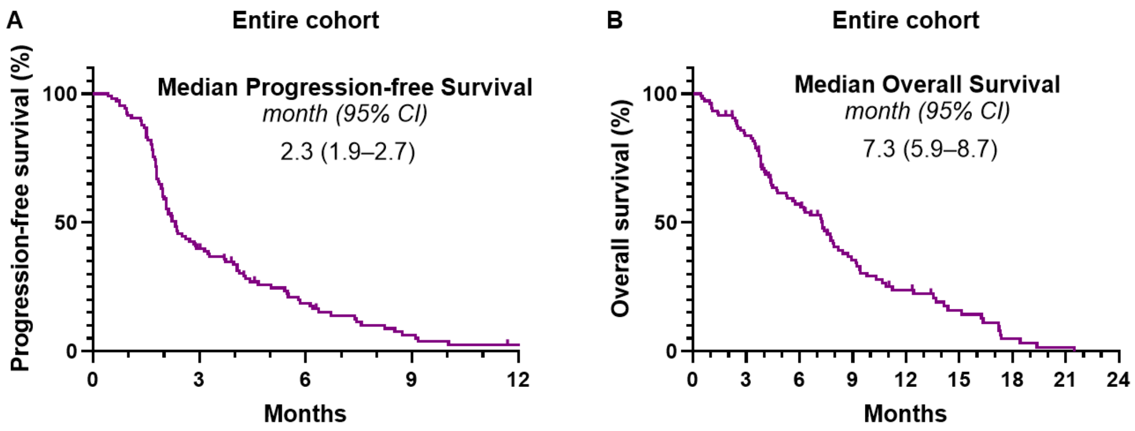

Figure S1. Survival outcomes of patients with metastatic colorectal cancer treated with trifluridine–tipiracil plus bevacizumab or trifluridine–tipiracil alone as salvage therapy. (A) Progression-free survival and (B) overall survival in the entire cohort.
